# Evolving geographic diversity in SARS-CoV2 and in silico analysis of replicating enzyme 3CL^pro^ targeting repurposed drug candidates

**DOI:** 10.1186/s12967-020-02448-z

**Published:** 2020-07-09

**Authors:** Nitin Chitranshi, Vivek K. Gupta, Rashi Rajput, Angela Godinez, Kanishka Pushpitha, Ting Shen, Mehdi Mirzaei, Yuyi You, Devaraj Basavarajappa, Veer Gupta, Stuart L. Graham

**Affiliations:** 1 Faculty of Medicine, Health and Human Sciences, Macquarie University, F10A, 2 Technology Place, North Ryde, NSW 2109 Australia; 2 Australian Proteome Analysis Facility, Macquarie University, North Ryde, NSW 2109 Australia; 3 School of Medicine, Deakin University, Melbourne, VIC Australia; 4 Save Sight Institute, Sydney University, Sydney, NSW 2000 Australia

## Abstract

**Background:**

Severe acute respiratory syndrome (SARS) has been initiating pandemics since the beginning of the century. In December 2019, the world was hit again by a devastating SARS episode that has so far infected almost four million individuals worldwide, with over 200,000 fatalities having already occurred by mid-April 2020, and the infection rate continues to grow exponentially. SARS coronavirus 2 (SARS-CoV-2) is a single stranded RNA pathogen which is characterised by a high mutation rate. It is vital to explore the mutagenic capability of the viral genome that enables SARS-CoV-2 to rapidly jump from one host immunity to another and adapt to the genetic pool of local populations.

**Methods:**

For this study, we analysed 2301 complete viral sequences reported from SARS-CoV-2 infected patients. SARS-CoV-2 host genomes were collected from The Global Initiative on Sharing All Influenza Data (GISAID) database containing 9 genomes from pangolin-CoV origin and 3 genomes from bat-CoV origin, Wuhan SARS-CoV2 reference genome was collected from GeneBank database. The Multiple sequence alignment tool, Clustal Omega was used for genomic sequence alignment. The viral replicating enzyme, 3-chymotrypsin-like cysteine protease (3CL^pro^) that plays a key role in its pathogenicity was used to assess its affinity with pharmacological inhibitors and repurposed drugs such as anti-viral flavones, biflavanoids, anti-malarial drugs and vitamin supplements.

**Results:**

Our results demonstrate that bat-CoV shares > 96% similar identity, while pangolin-CoV shares 85.98% identity with Wuhan SARS-CoV-2 genome. This in-depth analysis has identified 12 novel recurrent mutations in South American and African viral genomes out of which 3 were unique in South America, 4 unique in Africa and 5 were present in-patient isolates from both populations. Using state of the art in silico approaches, this study further investigates the interaction of repurposed drugs with the SARS-CoV-2 3CL^pro^ enzyme, which regulates viral replication machinery.

**Conclusions:**

Overall, this study provides insights into the evolving mutations, with implications to understand viral pathogenicity and possible new strategies for repurposing compounds to combat the nCovid-19 pandemic.

## Background

In early January 2020, the World Health Organisation (WHO) reported cases of pneumonia of an unknown cause in Wuhan City, Hubei Province of China, and by 30 January 2020, WHO escalated the warning to public health emergency of international concern. By 12 March 2020, the novel coronavirus (nCoV) outbreak achieved a global pandemic status and was recognised as novel Covid-19 disease (nCovid-19) [1]. The present coronavirus outbreak is associated with severe acute respiratory syndrome 2 (SARS-CoV-2), phylogeny and taxonomy designated [2]. Worldometer reported the total SARS-CoV-2 infected cases on 31 May 2020 as 6,238,550 and deaths 374,374 worldwide (https://www.worldometers.info/coronavirus/#countries). The pathogen has been established to transmit from human to human contact and has quickly spread to more than 187 countries across the globe (https://gisanddata.maps.arcgis.com/).

**Fig. 1:**
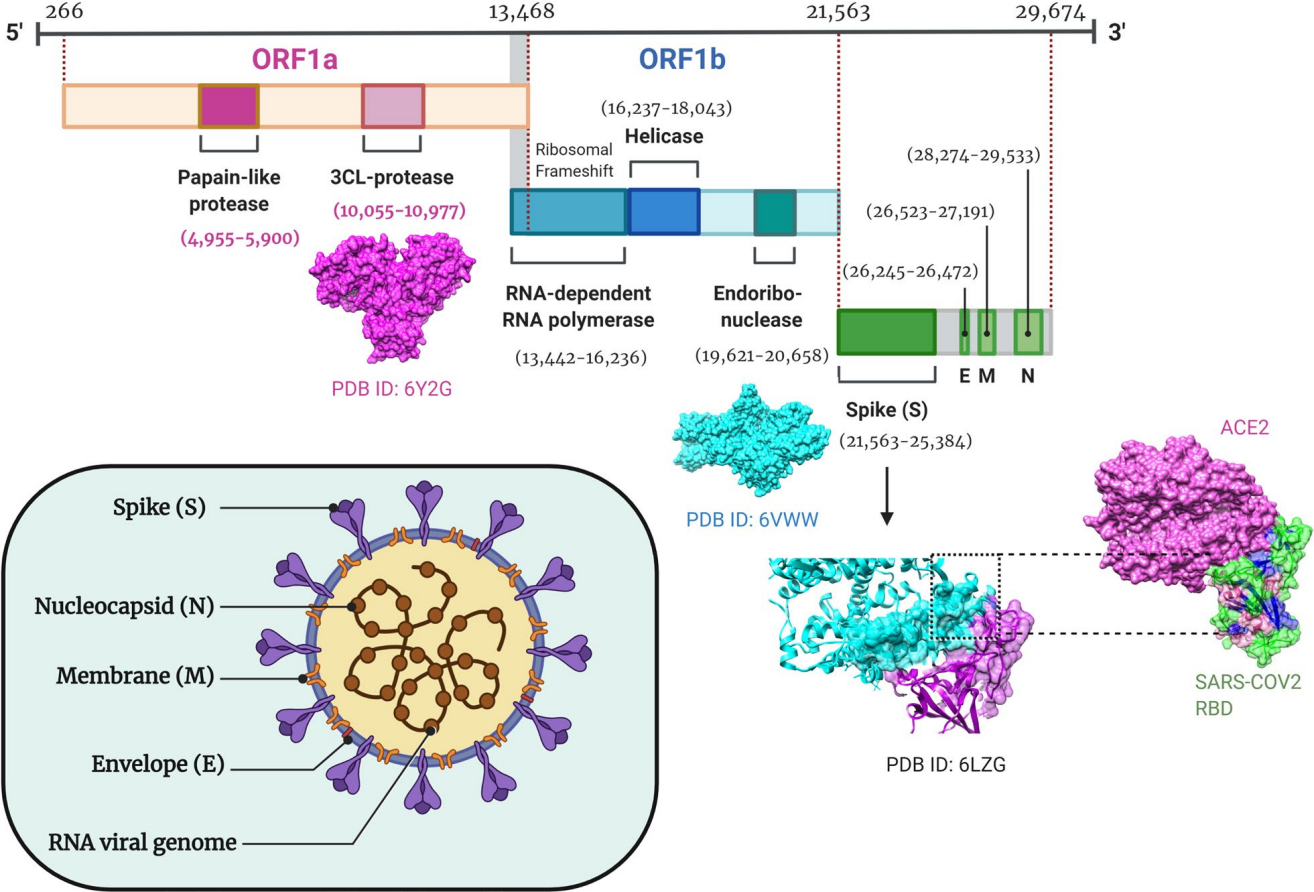
Schematic representation of SARS-CoV-2 structure showing single stranded RNA viral genomic assembly of 29,674 nucleotide base pair which encodes open reading frame 1a (ORF1a, nt 266–13,468), yellow colour, open reading frame 1b (ORF1b, nt 13,468–21,563), blue colour, Spike (S, nt 21,563–25,384), Envelope (E, nt 26,245–26,472), Membrane (M, nt 26,523–27,191) and Nucleocapsid (N, nt 28,274–29,533) proteins in green. ORF1a gene encodes papain-like protease and 3CL protease, ORF1b gene encodes RNA-dependent RNA polymerase, helicase and endo ribo-nuclease, S, E, M and N gene encodes spike, membrane glycoprotein and nucleocapsid phosphoprotein respectively. Three-dimensional crystal structure of 3CL-protese, endoribonuclease and SARS-Cov-2 spike protein receptor binding domain (RBD) engaged human angiotensin converting enzyme 2 (ACE2) receptor were collected from protein data bank

Coronaviruses are single and positive stranded RNA viruses belonging to the genus *Coronavirus* of the family Coronaviridae that can cause acute and chronic respiratory and central nervous system illnesses in animals, including in humans [3, 4]. The infection can also cause mild episodes of follicular conjunctivitis in certain patients. In animal models, the infection has been shown to induce anterior uveitis, retinitis, and optic neuritis like symptoms [5]. Recent study has shown formation of hyper-reflective lesions in the ganglion cell and inner plexiform layers of the retina particularly around the papillomacular bundles [6]. The disease has also been shown to affect sense of smell and taste bud sensitivity in patients [7]. All coronaviruses have a minimum of 3 basic viral proteins (i) an envelope protein (E), which is a highly hydrophobic protein involved in several aspects of the virus life cycle such as assembly and envelope formation [8] (ii) a spike protein (S), a glycoprotein involved in receptor recognition and membrane fusion [9] and (iii) a membrane protein (M), which plays a key role in virion assembly [10] (Fig. 1). The viral genome also encodes two open reading frames (ORF), ORFa and ORFb that activate intracellular pathways and triggers the host innate immune response [11]. The polyprotein encoded by the virus are initially processed by two main viral proteases, which include a papain-like cysteine protease (PL^pro^) and chymotrypsin-like cysteine protease, known as 3C-like protease (3CL^pro^), into intermediate and mature non-structural proteins [12].

The main proteinase 3CL^pro^, is one of the primary targets for development in an antiviral drug therapies, as it plays a critical role in the viral replication [13]. K11777, camostat and EST, are cysteine protease inhibitors, which have been shown to inhibit SARS-CoV 3CL^pro^ replication in cell culture conditions [14, 15]. Recent release of the high-resolution crystal structure for the main proteinase 3CL^pro^ (Protein Data Bank, PDB ID: 6Y2G), describing an additional amide bond with the α-ketoamide inhibitor pyridone ring to enhance the half-life of the compound in plasma [16] is suggested to accelerate the targeted drug discovery efforts. Two HIV-1 proteinase inhibitors, lopinavir and ritonavir, have been considered to target SARS-CoV [17]. Interestingly, the substrate binding cleft is located between domains I and II of both SARS-CoV 3CL^pro^ and SARS-CoV-2 3CL^pro^ enzymes [16, 18].

Since the initial stages of the SARS-CoV-2 outbreak, laboratories and hospitals around the world have sequenced viral genome data with unprecedented speed, enabling real-time understanding of this novel disease process, which will hopefully contribute to the development of novel candidate drugs. The complete genomes of SARS-Cov-2 from all over the world have been deposited at The Global Initiative on Sharing Avian Influenza Data (GISAID) [19] database and more sequences continue to be deposited with the passage of time. Development of a novel vaccine against SARS-CoV-2 so far remains elusive and requires a thorough understanding of molecular changes in viral genetics. This may be attained by freely accessing the GISAID database and processing the data to enhance our understanding of the fine biochemical and genetic differences that differentiate this virus from the previously known strains [20].

It is well known that viruses are non-living and that they require host cells to survive and to reproduce, with the sole aim to perpetuate themselves. When a virus jumps from animal to human, it is termed a zoonotic virus. This occurred during the SARS outbreak of 2002, when a new coronavirus spread around the world and resulted in death of hundreds of people [21]. In 2012, another novel coronavirus outbreak, termed Middle East respiratory syndrome (MERS), caused over 400 fatalities and spread to over 20 different countries [22]. There are currently many circulating viruses, but why SARS-CoV-2 has achieved such a devastating pandemic status and whether this pandemic will subside remain unanswered.

The purpose of this study is to characterise known viral variants that have spread across different countries, especially hot-spot regions, with a focus on recurrent mutations in South American and African geographical regions. We also focused on the SARS-CoV-2 main proteinase, 3CL^pro^ which is highly conserved in most of the coronaviruses and has been suggested to be a potential drug target to fight against nCovid-19. Repurposed drugs such as flavonoids and biflavanoids, known anti-malarial and anti-viral drugs and the inhibitory effects of vitamins could selectively inhibit this enzyme and can be used either alone or in combination with other disease management approaches to suppress the virulence of SARS-CoV-2. These bioinformatics, computational modelling and molecular docking approaches using repurposed drugs could be particularly useful in the current nCovid-19 outbreak.

## Methods

### Collection of SARS-Cov-2 genome

The Global Initiative on Sharing Avian Influenza Data (GISAID) is headquartered in Munich, Germany and is a public–private partnership project between German government and the non-profit organization founded by leading medical researchers in 2006. Since December 2019, GISAID has become a repository storage database for nCovid-19 genome. The genome analysis was carried out for data deposited up to 31 May 2020 (https://www.gisaid.org/). Severe acute respiratory syndrome coronavirus 2 (SARS-CoV-2) Wuhan genome was collected from NCBI, NC_045512.2.

### Multiple sequence alignment and Phylogenetic tree construction

Multiple sequence alignment (MSA) of all nucleotide sequences were carried out in the EMBL-EBI Clustal Omega server to investigate sequence conservation [23, 24]. The Newick format for the multiple align sequence was used to generate phylogeny [25]. The phylogenetic tree was constructed in the Interactive Tree of Life (iTOL) online tool [26]. The iTOL server generate phylogeny trees in a circular (radial) and normal standard trees. The circular trees can be rooted and displayed in different arc sizes [27–29].

### Structure analysis SARS and SARS-CoV-2 3CL^pro^

Crystal structure of SARS and SARS-CoV-2 3CL^pro^ with bound inhibitors were collected from the protein data bank (PDB) [30]. PDB ID: 3TNT, SARS main protease was selected as reference to analyse the variants in SARS-CoV-2 3CL^pro^ (PDB ID: 6Y2G). All the PDBs were visualised using UCSF Chimera software [31]. Multiple alignment, ribbon, surface and superimposition module in Chimera software were used for analysis and image generation [24, 32].

### Computer aided molecular modelling

#### Collection and preparation of SARS-CoV-2 protease inhibitors

The dataset comprises of flavones and biflavanoids, anti-viral, anti-malarial and vitamins as SARS-CoV-2 3CL^pro^ inhibitors [16]. In total 17 repurposed drugs were collected from the Pubchem database [33]. Two-dimensional (2D) structures were downloaded from the Pubchem database in.sdf format. The inhibitor energies were minimized using the Austin Model-1 (AM1) until the root mean square (RMS) gradient value became smaller than 0.100 kcal/mol Å and later re-optimization was done by MOPAC (Molecular Orbital Package) method [34, 35]. Later, all the inhibitors were converted to.pdb format in Open Babel software [36] and submitted to molecular docking studies.

#### Selection and preparation of SARS-Cov-2 main protease protein (3CL ^pro^)

Crystal structure of the SARS-CoV-2 3CL^pro^ was retrieved from PDB (PDB ID: 6Y2G). The protein macromolecule (SARS-CoV-2 3CL^pro^) optimization was carried out in UCSF Chimera software [31, 37, 38] by adding polar hydrogen atoms, removing water molecules, implying amber parameters, followed by minimization with the MMTK method in 500 steps with a step size of 0.02 Å. SARS-CoV-2 3CL^pro^ contained chain A and B of 306 amino acids sequence length. Chain A of PDB ID: 6Y2G containing alpha-ketoamide (O6K) inhibitor was used for identification of substrate binding site.

### SARS-Cov-2 main protease protein (3CL ^pro^) inhibitors docking studies

The docking of SARS-CoV-2 3CL^pro^ specific pharmacological inhibitors into the catalytic site was performed by the AutoDock 4.2 program [39]. The alpha-ketoamide (O6K) inhibitor was extracted from the SARS-CoV-2 3CL^pro^ protein. The polar hydrogen atoms were added, the non-polar hydrogen atoms were merged, Gasteiger charges were assigned and solvation parameters were added to the protease, SARS-CoV-2 3CL^pro^ protein. The protonation state for all inhibitors and O6K were set to physiological pH and rotatable bonds of the ligands were set to be free. The AutoGrid program was also used to generate grid maps. Cys145 residue in the SARS-CoV-2 3CL^pro^ protein was selected with grid box dimensions of 40 × 40 × 40 Å formed around the Cys145 protease residue, which is present in the substrate binding site. Protein rigid docking was performed using the empirical free energy function together with the Lamarckian genetic algorithm (LGA) [40]. LGA default parameters were used in each docking procedure and 10 different poses were calculated. Chimera and Discovery Studio (DS) Visualizer2.5 [31] software were used for visualisation and calculation of protein–ligand interactions.

## Results

### Distribution analysis of SARS-CoV-2 in different geographic regions

**Fig. 2:**
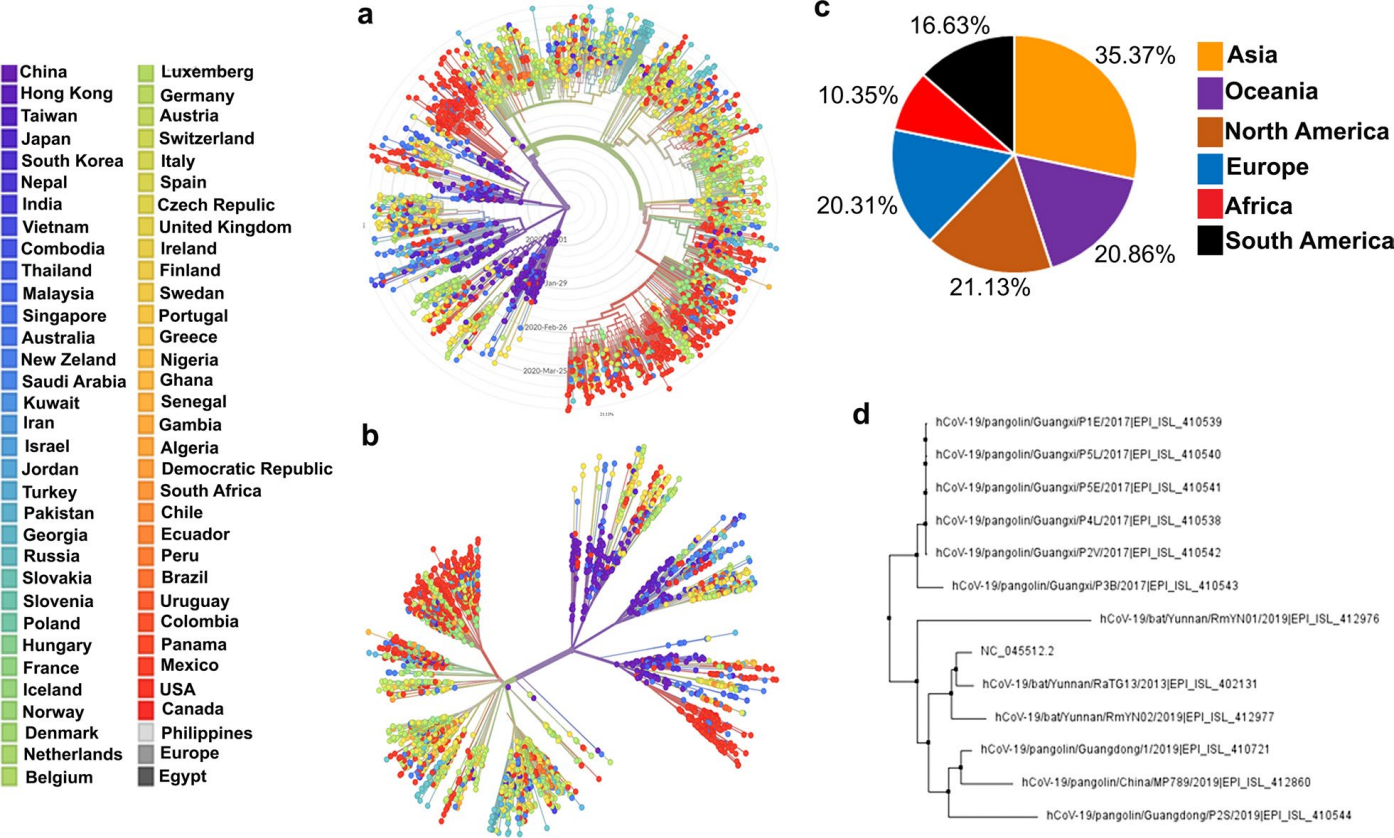
(**a**, **b**) Phylogenetic evolutionary relationships of SARS-CoV-2 virus showing an initial emergence in Wuhan, China, in Nov-Dec 2019 followed by continued human-to-human transmission. SARS-CoV-2 patient genome sequences deposited in GISAID database from more than 60 different countries (**a**) radial and (**b**) unrooted phylogeny created by Nextstrain program [66] (**c**) Pie chart representation of number of SARS-CoV-2 patient genomes deposited in GISAID till 15th April 2020 from six different regions; Asia (orange, 35.37%), Oceania (purple, 20.86%), North America (brown, 21.13%), Europe (blue, 20.31%), Africa (red, 10.35%) and South America (black, 16.63%). (**d**) Phylogenetic relationship of CoVs based on whole genome nucleotide sequences from bat, pangolin, and Wuhan SARS-CoV-2 (NC_045512.2) confirms that SARS-CoV-2 share > 90% similarity with bat SARS-CoV while pangolin could be the closest ancestral

A total of 9761 SARS-CoV-2 genomes were retrieved from The GISAID database (https://www.gisaid.org) that contain 3 sequences from bat (*Betacoronavirus*) and 9 sequences from Malayan Pangolin (Manis javanica) (Additional file 1: Table S1). Out of 9761 genome sequences, 2301 complete genome sequences of SARS-CoV-2 were selected randomly, aligned and compared with Wuhan SARS-CoV-2 (NC_045512.2) reference genome. We have divided our dataset into 6 different geographic areas: Europe (20.31%), North America (21.13%), Asia (35.37%), Oceania (20.86%), South America (16.63%) and Africa (10.35%). The European group comprises of SARS-CoV-2 infected patient data from the following countries: Austria, Belgium, Czech Republic, Denmark, Estonia, Finland, France, Germany, Greece, Hungary, Iceland, Ireland, Italy, Latvia, Lithuania, Luxembourg, Netherlands, Poland, Portugal,, Slovakia, Slovenia, Spain, Sweden, Switzerland, and United Kingdom. The North American group contains genomes from the United States and Canada. The Asian group comprises genomes obtained from patients located in China, Indonesia, Pakistan, Philippines, Taiwan, Turkey, Kuwait, Georgia, South Korea, Japan, Iran, India, Thailand, Hong Kong, Malaysia, Singapore, and Vietnam. The Oceanian group comprises genomes from Australia and New Zealand. South America includes Brazil, Peru, Chile, Colombia, Argentina, and Ecuador (Fig. 2a–c).

Sequences from bat-SARS-CoV and Pangolin-SARS-CoV were aligned and compared to the Wuhan SARS-CoV-2 (NC_045512.2) as a reference genome. To determine the evolutionary relationship among bat-CoV, Pangolin-CoV and SARS-CoV-2, we estimated a phylogenetic tree based on the nucleotide sequences of the whole-genome sequence. Bat-SARS-CoV and SARS-CoV-2 were grouped together and were observed to share > 96% similarity, whereas the Pangolin-SARS-CoV was closest evolutionary ancestor (Fig. 2d). Isolate of human Wuhan SARS-CoV-2 (NC_045512.2) shared 85.98% identity with Pangolin-SARS-CoV which suggests that Pangolin may be associated with SARS-CoV-2 evolution or subsequent outbreak [41, 42].

### Identification of hotspot mutations in SARS-Cov-2 complete genome from South American and African regions and analysis of main protease (3CL^pro^) sequence

**Fig. 3:**
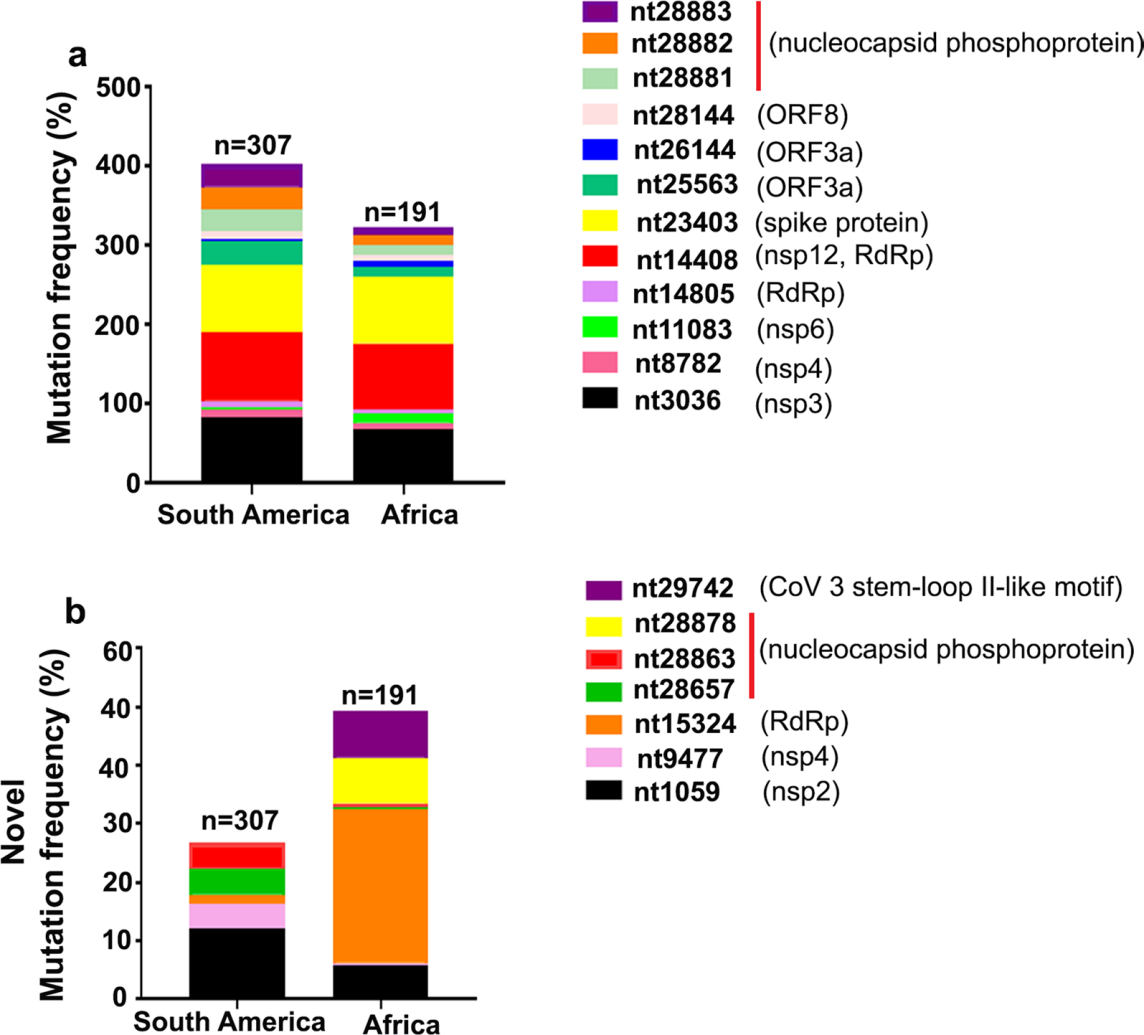
Graphical representation of SARS-CoV-2 mutation frequency in South American and African patient isolates. **a** Five novel recurrent hotspots mutations (namely 14,805, 25,563, 26,144, 28,882 and 28,883) were subdivided into 2 geographical areas: South America (n = 307) and Africa (n = 191). Previously confirmed mutations at positions nt3036, nt8782, nt11083, nt14408, nt23403, nt28144 and nt28881 were also present in South American and African populations. We normalize the mutation frequency percentage by estimating the frequency of genomes carrying mutation and comparing it with the overall number of collected genomes per geographical area. The graph shows the cumulative mutation frequency of all given mutations present in South American and African regions. Mutation localisation in viral genes are reported in the legend as well as the proteins (i.e. non-structural protein, nsp) presenting these mutations. **b** It is also evident that South American and African clusters show a differential pattern of novel mutations: mutation 1059 (black), 9477 (pink), 28,657 (green) and 28,878 (red) in South American, whereas mutation 1059 (black), 15,324 (orange), 28,878 (yellow) and 29,742 (magenta) are present with greater frequency in African patients

Recently, Pachetti et al. [43] has reported eight novel recurrent mutations of SARS-Cov-2 that have been identified in positions 1397, 2891, 14,408, 17,746, 17,857, 18,060, 23,403 and 28,881 in Asian, Oceanic, European and North American outbreaks. However, SARS-CoV-2 mutations from South American and African patient isolates are not yet reported. We confirmed the occurrence of these mutations in South Americans and Africans located at positions 3036, 8782, 11,083,14,408, 23,403, 28,144 and 28,881 as reported in previous literature [43]. Our study highlights the presence of additional “conserved mutations” in the South American and African communities, considering only those occurring ≥ 5 times in our database. We report here 12 new mutations that have evolved in the SARS-Cov2 sequence in South American and African populations. These are located at positions 14,805, 25,563, 26,144, 28,882, 28,883, 9477, 28,657, 28,863, 1059, 15,324, 28,878 and 29,742 sites. The high tendency of the virus to demonstrate genetic variability is evident from the fact that even within these variants, three variations 9477 (nsp4), 28,657 and 28,863 (ORF9, structural protein) were uniquely identified in isolates from South American patients while four novel mutations viz. 1059 (nsp2), 15,324 (RdRp), 28,878 (ORF9, structural protein), and 29,742 (stem-loop II-like motif) were detected only in isolates from African patient samples (Fig. 3b). Interestingly, some mutations were identified to be common between these two separate sets of sequences that have been reported from the two distinct geographical locations viz. 14,805, 25,563, 26,144, 28,882 and 28,883, belonging to gene ORF1ab (14,805 RNA-dependent RNA polymerase (RdRp), ORF3a (25,563 and 26,144 ORF3a protein) and ORF9, N gene (28,882 and 28,883 nucleocapsid phosphoprotein) sequences, respectively (Fig. 3a). An interesting finding of this analysis is the concurrence of 14,805 mutation with 14,808 mutation in the same locus. This double point mutation was observed in RdRp genome from isolates of both South American and African patients. In contrast, 28,882/28,883 mutation locus corresponded with another previously reported mutation 28,881, and this triple point mutation was also present in both the South American and African genomic sequences. Identification of point mutations at the same locus indicates the high susceptibility of these genetic regions to change as the virus evolves.

For its actions, single-stranded SARS-CoV-2 RNA viral genome encodes two protease polyproteins (i) papain-like cysteine-protease (PL^pro^) and (ii) the chymotrypsin-like cysteine protease known as 3C-like protease (3CL^pro^). 3CL^pro^, which is a main protease and therefore important in order to examine the incidence of any mutation in SARS-CoV-2 3CL^pro^. Multiple sequence alignment of the SARS-CoV-2 genome collected from patients in six different geographical locations exhibited 100% similarity and no discernible variations in sequences obtained from diverse geographical regions, for this enzyme.

### SARS-CoV and SARS-CoV-2 similarity

**Fig. 4:**
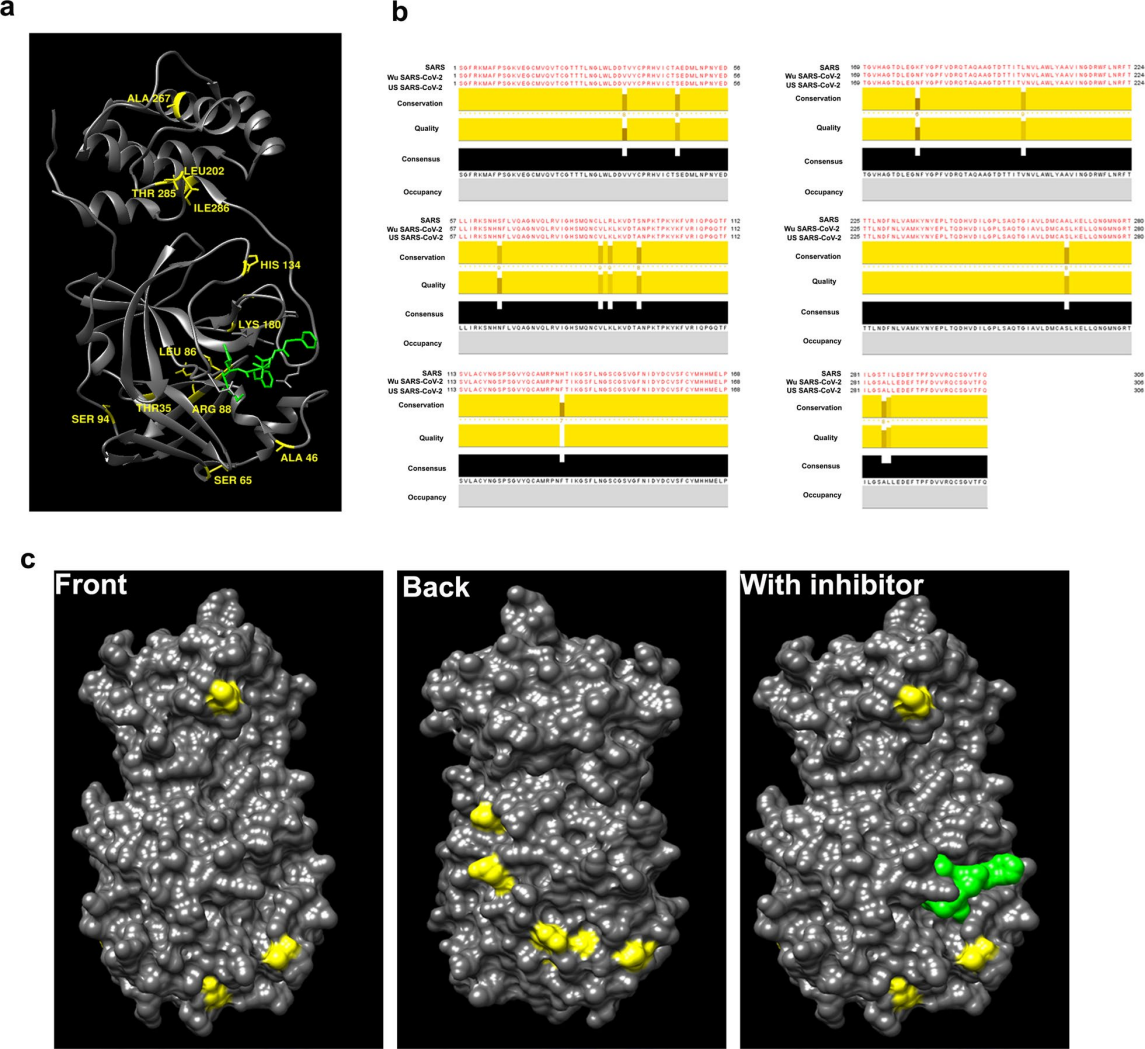
SARS-CoV-2, Main proteinase 3CL^pro^ analysis. **a** Cartoon representation structure of the SARS-CoV-2 3CL^pro^ homodimer with inhibitor (green) in greyish black colour. Variant positions of amino acids in 3CL^pro^ (Thr35Val, Ala46Ser, Ser65Asn, Leu86Val, Arg88Lys, Ser94Ala, His134Phe, Lys180Asn, Leu202Val, Ala267Ser, Thr285Ala and Ile286Leu) are shown in yellow colours. **b** Multiple sequence alignment between SARS-CoV and SARS-CoV-2 3CL^pro^ from Wuhan (Wu) and United States of America (US) patients sharing more than 90% sequence identity. **c** Surface view representation of SARS-CoV-2 3CL^pro^ (PDB ID: 6Y2G) showing muted amino acid residues in yellow and alpha-ketoamide inhibitor (green) in the substrate binding region. Images are generated by UCSF Chimera software

SARS and SARS-CoV-2 complete genomes were collected from NCBI, GenBank database (NC_004718 and NC_045512). Protease nucleotide sequences were extracted from SARS (NC_004718) and were aligned with SARS-CoV-2 (NC_045512). Clustal Omega alignment of 918 SARS nucleotides showed around 95% similarity with SARS-CoV-2 (Additional file 2: Table S2). Higher amino acid sequence identity was also observed in SARS-CoV and SARS-CoV-2 main protease (3CL^pro^) derived from Wuhan and US patients. SARS-CoV and SARS-CoV-2 3CL^pro^ showed highly conserved region in both the catalytic sites, His41 and Cys145 [44] and substrate binding region of the enzyme (163-167 and 187-192) [45] (Fig. 4a), inferring that these proteases exhibit high similarities. Furthermore, 12 variant positions (Thr35Val, Ala46Ser, Ser65Asn, Leu86Val, Arg88Lys, Ser94Ala, His134Phe, Lys180Asn, Leu202Val, Ala267Ser, Thr285Ala and Ile286Leu) were observed in SARS-CoV-2 3CL^pro^ (Fig. 4b, c). The effects of mutations and potential resultant amino acids on SARS-CoV-2 3CL^pro^ structure are expected to conserve the polarity and hydrophobicity, except when the resulting amino acid is Leucine at 286 position. However, it is important to mention that these 12 variants are not present in catalytic and substrate binding regions which are involved in critical proteolytic activity of the SARS-CoV-2 protease molecule.

### Docking study of SARS-CoV-2 3CL^pro^ inhibitors

**Fig. 5:**
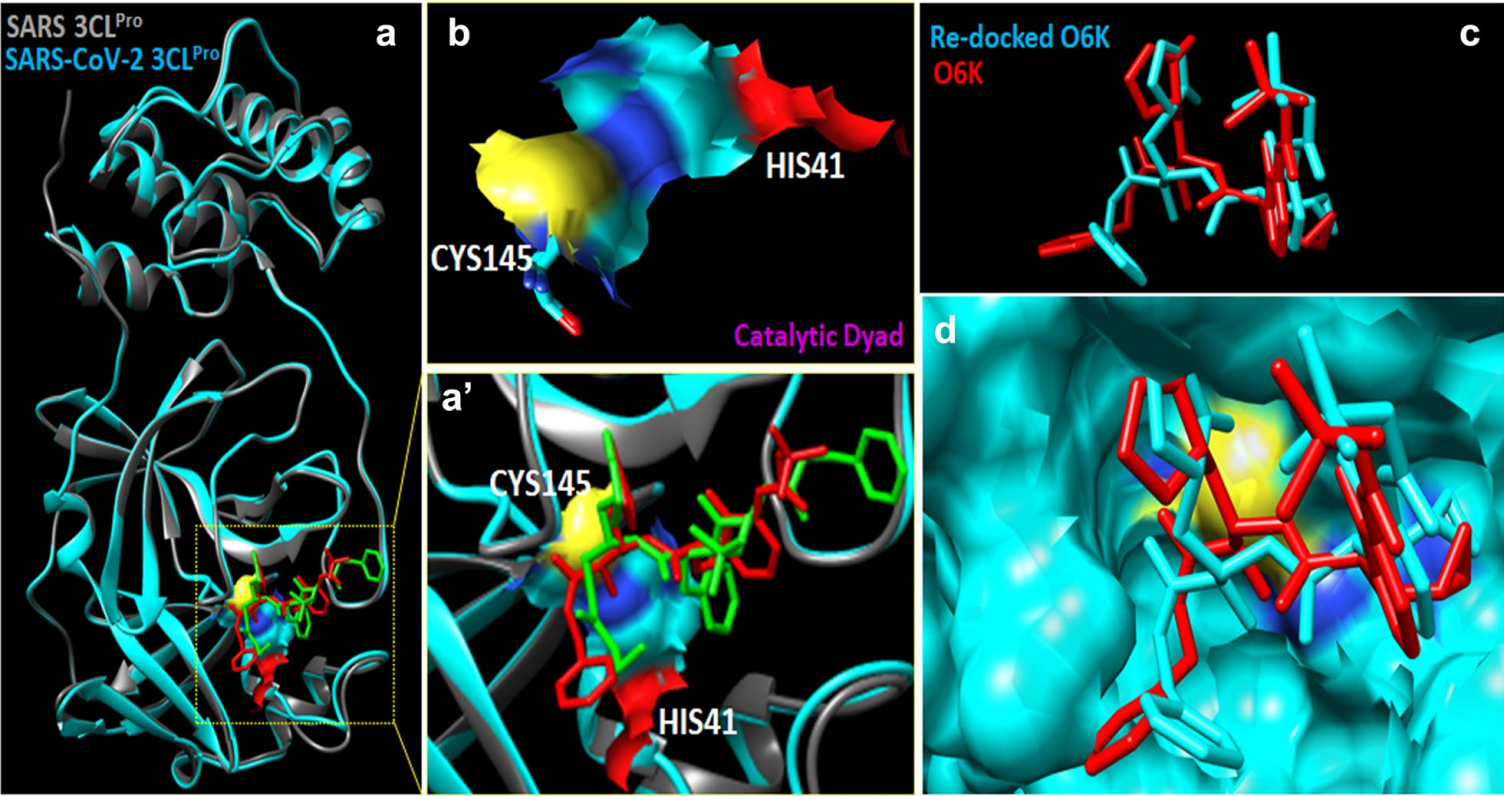
**a** Cartoon representation of superimposed structures from SARS-CoV 3CL^Pro^ (PDB ID: 3TNT, grey) and SARS-CoV-2 3CL^pro^ (PDB ID: 6Y2G, cyan) showing 94.44% sequence identity. Two different 3CL^pro^ inhibitors represented in red and green colour in the substrate binding region (**a**’) magnified view of substrate binding region. **b** The residues of the catalytic dyad (His41 and Cys145) are shown in surface view. Autodock 4.2 docking protocol was validated by re-docking of O6K inhibitor in SARS-CoV-2 3CL^pro^, original O6K inhibitor is shown in red colour and re-docked pose in cyan colour **c** three-dimensional and **d** surface view representation

The SARS-CoV-2 3CL^pro^ receptor binding pocket was determined by superimposing SARS and SARS-CoV-2 3CL^pro^ with their respective inhibitors (Fig. 4). Interestingly, Needleman-Wunsch alignment algorithm and BLOSUM-62 matrix analysis revealed 94.44% sequence identity between SARS (Fig. 5a, grey) and SARS-CoV-2 3CL^pro^ (Fig. 5a, Cyan). Cys-His catalytic dyad (Cys145 and His41) comprises the active catalytic binding site in SARS-CoV-2 3CL^pro^ (Fig. 5a’, b) and indicated the strong possibility that intended pharmacological inhibitors of SARS-CoV-2 3CL^pro^ may also suppress the activity of SARS-CoV-2 3CL^pro^ viral enzymes. Docking protocol for the Autodock 4.2 program was optimized by extracting and re-docking the alpha-ketoamide inhibitor named O6K in the binding pocket of SARS-CoV-2 3CL^pro^. The lowest binding energy − 6.45 kcal/mol and 18.72 µM inhibitory constant (K*i*) was predicted for alpha-ketoamide inhibitor (shown in Table 1). Re-docking of O6K inhibitor occupied the similar docking pose in the SARS-CoV-2 3CL^pro^ catalytic dyad active site as previously reported in the crystal structure (PDB ID: 6Y2G) (Fig. 5c, d).

**Table 1:** Dataset of 20 repurposed drugs and reference ligand (O6K) corresponding energies obtained from the docking test performed using AutoDock 4.2 program

S. no	C. name	BE^e^ (kcal/mol)	Ki (µM)	IME^e^ (kcal/mol)	V_dw_-H_b_-D_s_ (kcal/mol)	E^e^ (kcal/mol)	IE^e^ (kcal/mol)	TFE^e^ (kcal/mol)	USE	RMSD (Å)
1	O6K	− 6.45	18.72	− 10.33	− 10.36	+ 0.03	− 2.10	+ 3.88	− 2.10	5.59
	7, DHF	− 6.24	26.49	− 7.14	− 6.83	− 0.31	− 1.45	+ 0.89	− 1.45	32.85
2	Apigenin	− 7.52	3.05	− 8.75	− 8.52	− 0.20	+ 9.72	+ 1.19	+ 9.72	33.64
3	Luteolin	− 5.59	80.53	− 7.08	− 6.78	− 0.29	− 1.84	+ 1.49	− 184	33.26
4	Quercetin	− 6.16	30.49	− 7.95	− 7.56	− 0.39	− 1.96	+ 1.79	− 1.96	32.79
5	Amentoflavone	− 8.49	0.59	− 11.18	− 10.97	− 0.21	− 3.34	+ 2.68	− 3.34	35.22
6	Bilobetin	− 8.29	0.83	− 10.98	− 10.68	− 0.30	− 4.30	+ 2.68	− 4.30	35.32
7	Ginkgetin	− 8.14	1.09	− 10.82	− 10.52	− 0.30	− 3.51	+ 2.68	− 3.51	34.77
8	Chloroquine	− 4.95	233.3	− 7.34	− 6.96	− 0.38	− 1.34	+ 2.39	− 1.34	30.78
9	Hy-chloroquine	− 5.77	58.47	− 8.76	− 8.53	− 0.23	− 0.68	+ 2.98	− 0.68	30.56
10	Artemisinin	− 6.40	20.22	− 6.70	− 6.50	− 0.20	+ 0.06	+ 0.30	+ 0.06	31.94
11	Remdesivir	− 6.40	28.28	− 11.47	− 11.55	+ 0.07	− 4.52	+ 5.07	− 4.52	31.05
12	Darunavir	− 7.16	5.64	− 11.34	− 11.26	− 0.08	− 3.75	+ 4.18	− 375	33.98
13	Lopinavir	− 6.98	7.68	− 11.75	− 11.57	− 0.18	− 4.92	+ 4.77	− 4.92	29.98
14	Galidesivir	− 4.69	362.43	− 6.48	− 5.79	− 0.70	− 2.02	+ 1.79	− 2.02	32.04
15	Favipiravir	− 4.15	905.42	− 4.45	− 4.31	− 0.14	− 0.09	+ 0.30	− 0.09	32.27
16	Ritonavir	− 7.45	3.49	− 13.11	− 13.15	+ 0.03	− 3.46	+ 5.67	− 5.67	28.61
17	Umifenovir	− 5.71	65.42	− 8.39	− 8.13	− 0.26	− 1.81	+ 2.68	− 1.81	31.86
18	Vitamin C	− 4.22	805.2	− 6.01	− 5.72	− 0.29	− 2.03	+ 17.79	− 2.03	29.14
19	Vitamin D	− 7.75	2.08	− 9.84	− 9.78	− 0.06	− 1.75	+ 2.09	− 1.75	33.21
20	Vitamin E	− 7.59	2.75	− 11.46	− 11.40	− 0.06	− 1.57	+ 3.88	− 1.57	25.17

*BE*^*e*^ estimated binding free energy in kcal mol^−1^; *Ki* inhibitory constant in micro-molar; *IME*^*e*^ final Intermolecular energy in kcal mol^−1^; *V*_*dw*_–*H*_*b*_–*D*_*s*_ Van der waals-hydrogen bond-desolvation energy component of binding free energy in kcal mol^−1^; *E*^*e*^ electrostatic energy in kcal mol^−1^; *IE*^*e*^ final total internal energy in kcal mol^−1^; *TFE*^*e*^ torsional free energy in kcal mol^−1^; *USE* unbound system’s energy; *RMSD* root mean square deviation (Å)

**Fig. 6:**
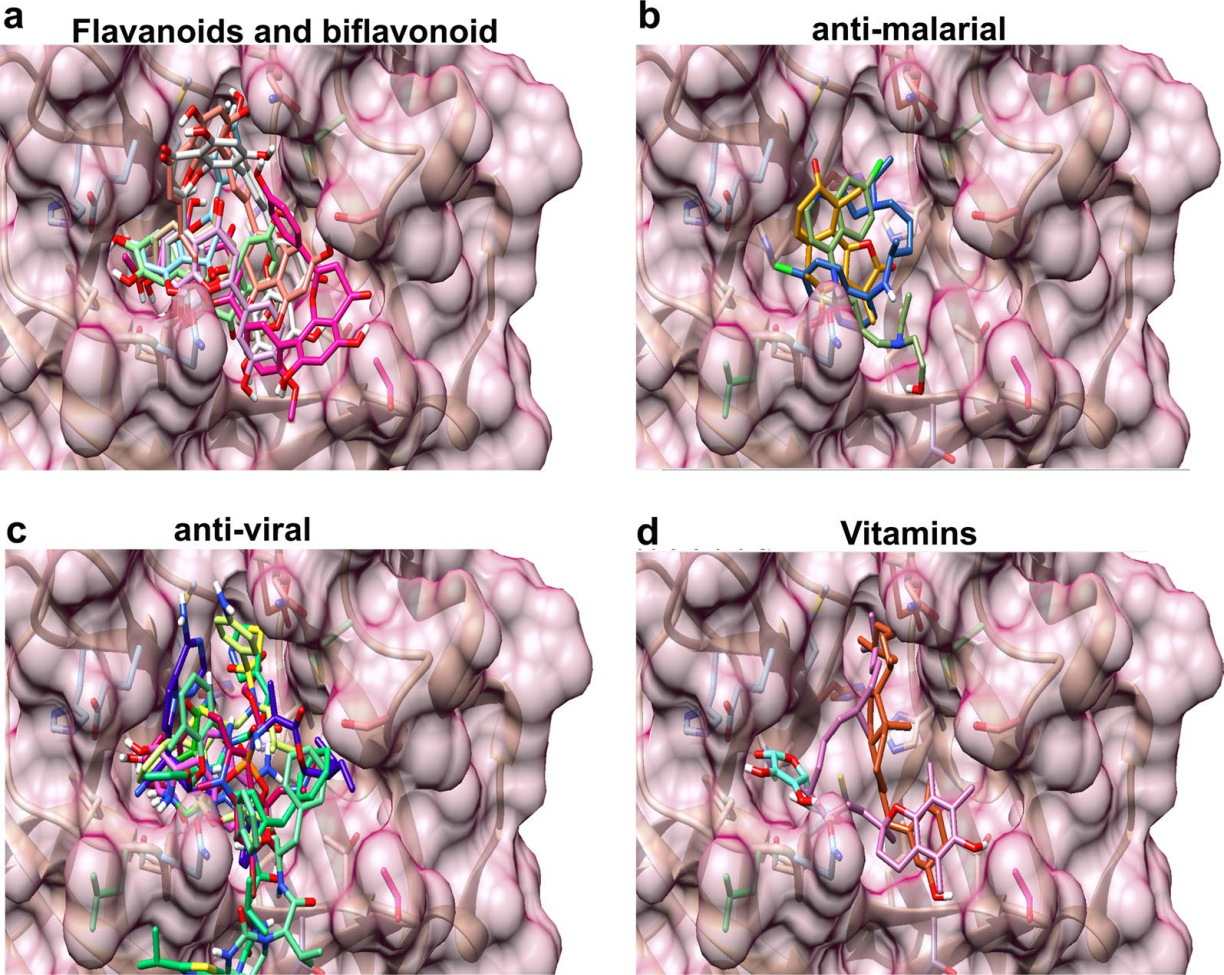
Binding modes of different repurposed drugs in the substrate binding region of SARS-CoV-2 3CL^pro^ (**a**) flavanoids and biflavonoid (7,8 DHF (purple), apigenin (pink), luteolin (orange), quercitin (green), amentoflavone (grey), bilobetin (brown) and ginkgetin (white) (**b**) anti-malarial (chloroquine (green), hydroxychloroquine (golden yellow) and artemisinin (blue) (**c**) anti-viral remdesivir (yellow), darunavir (blue), lopinavir (red), galidesivir (dark pink), favipiravir (light blue), ritonavir (light pink) and umifenovir (green) and **d** vitamins [vitamin C (cyan), vitamin D (golden orange) and vitamin E (pink)]

Seven flavonoids and biflavonoid, three anti-malarial compounds, seven anti-viral drugs and three vitamin molecules were subjected to automated docking within the active site of SARS-CoV-2 3CL^pro^ catalytic-dyad. The superimposition of all docked flavones and biflavones (Fig. 6a), anti-malarial drugs (Fig. 6b), anti-viral drugs (Fig. 6c) and vitamins (Fig. 6d) are shown in Fig. 6 and various binding parameter have been tabulated in detail in Table 2.

**Table 2:** Repurposed drugs (20) and O6K inhibitor interactions with the SARS-Cov-2 3CL^pro^ key amino acid residues involved in replication activity

S. no	Compound name/PubChem CID	H-bond residue
	O6K	His41, Phe140, Cys145, His163, His164, Glu166
1	7, DHF (PubChem CID:1880)	Ser144, His163
2	Apigenin (PubChem CID:5280443)	Cys145, His163
3	Luteolin (PubChem CID:5280445)	Thr26, His163, Glu166
4	Quercetin (PubChem CID:5280343)	Phe140, Leu141, Cys145
5	Amentoflavone (PubChem CID:5281600)	Thr26, His41, Ser46, Ser144, Cys145, Glu166
6	Bilobetin (PubChem CID:5315459)	Thr25, Leu141, Ser144, Cys145
7	Ginkgetin (PubChem CID:5271805)	Thr26, Gly143, His163
8	Chloroquine (PubChem CID:2719)	His41, Gly143, Cys145
9	Hydroxychloroquine (PubChem CID:3652)	Thr26, Asn142, Glu166
10	Artemisinin (PubChem CID:68827)	His41, Leu141, Asn142, Gly143, Ser144, Glu166
11	Remdesivir (PubChem CID:121304016)	Ser46, Ser144, Cys145, His163
12	Darunavir (PubChem CID:213039)	Gly143, Ser144, Cys145, His164
13	Lopinavir (PubChem CID:92727)	Thr26, His41, Gly143, Ser144, Cys145
14	Galidesivir (PubChem CID:10445549)	Phe140, Leu141, Gly143, Ser144, Cys145, His163, Glu166
15	Favipiravir (PubChem CID:492405)	Ser144, His163, His164, Glu166
16	Ritonavir (PubChem CID:392622)	Thr26, His41, Cys145
17	Umifenovir (PubChem CID:131411)	Thr26
18	Vitamin C (PubChem CID:54670067)	Asn142, Ser144, Cys145, His163, Gln166
19	Vitamin D (PubChem CID:5280795)	Thr24, Thr26, His41, Cys145
20	Vitamin E (PubChem CID:14985)	Thr25, Cys145

**Fig. 7:**
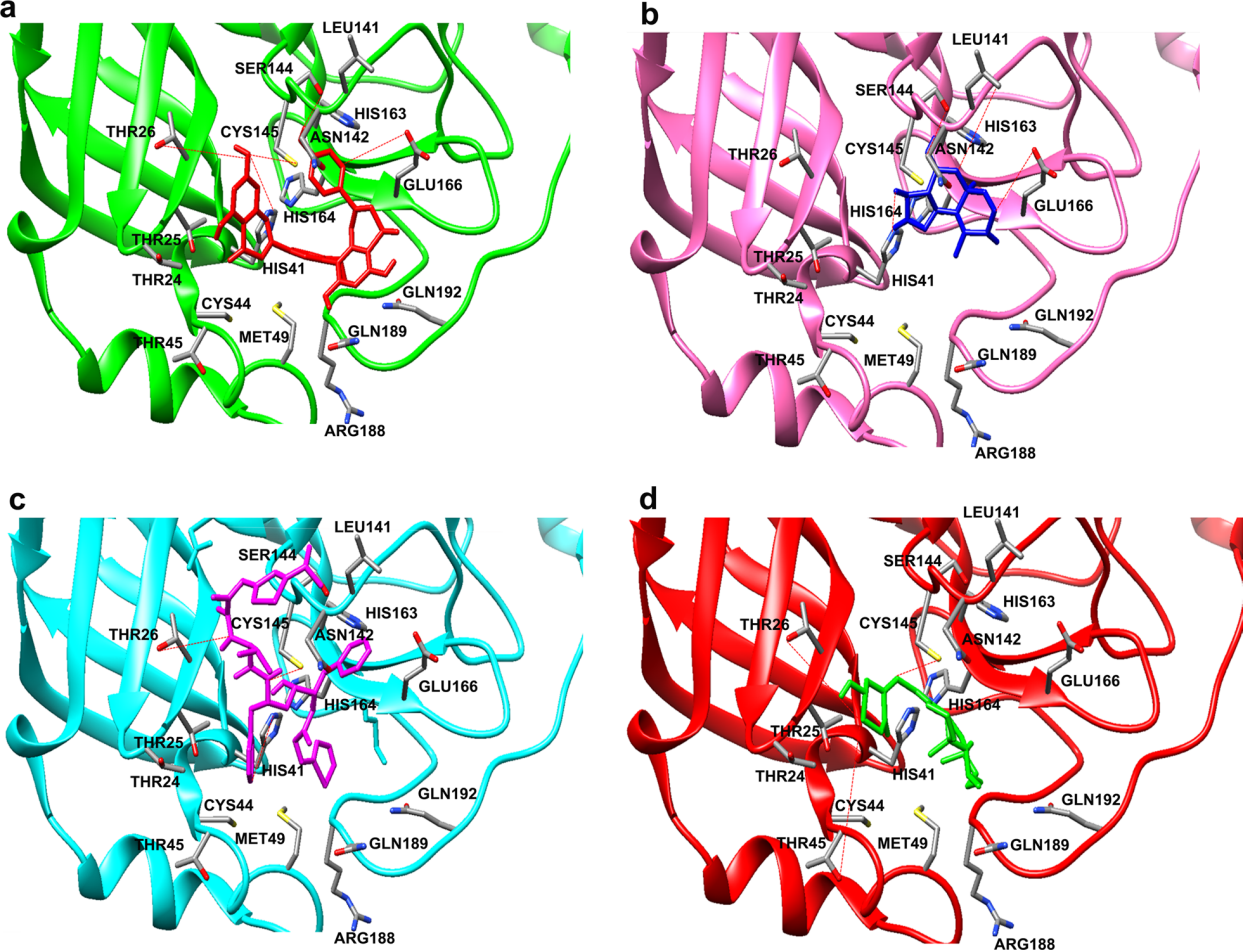
The binding model of repurposed drugs against SARS-CoV-2 3CL^pro^. **a** Binding mode of the Amentaflavone drug (red) in SARS-CoV-2 3CL^pro^ (green) substrate binding pocket. **b** Artemisinin drug (blue) binding mode in SARS-CoV-2 3CL^pro^ (pink) substrate binding pocket. **c** Binding mode of the Ritonavir drug (magenta) in SARS-CoV-2 3CL^pro^ (cyan) substrate binding pocket. **d** Binding mode of the Vitamin D (green) in SARS-CoV-2 3CL^pro^ (red) substrate binding pocket. Protein–ligand interaction (hydrogen bond) are shown with red dotted lines

Amentaflavone, a biflavonoid showed the highest binding energy (− 8.49 kcal/mol) implicating a strong affinity with SARS-CoV-2 3CL^pro^. This corresponded with previously reported enzyme inhibitory assays with amentaflavone that showed the highest IC_50_ value at low concentrations of the molecule, 8.3 ± 1.2 µM [46]. However, bilobetin demonstrated the lowest IC_50_ value at a higher concentration of 72.3 ± 4.5 µM in SARS-CoV enzyme activity assays [46]. In contrast, our docking studies revealed that bilobetin, predicted almost comparable binding energy with that of amentaflavone (− 8.29 kcal/mol) suggesting that mutation in SARS-CoV-2 3CL^pro^ could potentially disrupt hydrogen bonding or induce some conformational change that could result in alterations in the binding site thus affecting inhibitor interactions with the enzyme active site residues. Amentaflavone showed H-bond interactions with the catalytic dyad residues (Cys145 and His41) as well as noteworthy interactions with the SARS-CoV-2 3CL^pro^ residues Thr26, Ser46, Ser144 and Glu166 whereas His164, and Gln189 amino acids contributed to the hydrophobic interactions for the SARS-CoV-2 3CL^pro^ inhibitors (Fig. 7a).

Three antimalarial drugs were then selected to study their inhibitory actions on SARS-CoV-2 3CL^pro^. We found, Artemisinin, a natural compound derived from Chinese herb Artemisia *annua* produces the highest docking score (− 6.40 kcal/mol) as compared to O6K, chloroquine (-4.95 kcal/mol) and hydroxychloroquine (− 5.77 kcal/mol) anti-malarial molecules. Importantly, Artemisinin has demonstrated broad anti-viral activity against human cytomegalovirus, herpes simplex virus type 1, Epstein-Barr virus, hepatitis B virus, hepatitis C virus, and bovine viral diarrhea virus [47]. Artemisinin was shown to exhibit hydrogen bonding with His41, Leu141, Asn142, Gly143, Ser144 and Glu166 SARS-CoV-2 3CL^pro^ amino-acid residues (Fig. 7b).

Amongst the seven antiviral drugs, Ritonavir showed the highest binding energy (-7.45 kcal/mol) and lowest inhibitory constant K*i* value (3.49 µM). Ritonavir produced hydrogen bond interactions with Thr26, His41 and Cys145 SARS-CoV-2 amino acids (Fig. 7c). A combination of two HIV-1 protease inhibitors, lopinavir and ritonavir, were given to critically ill SARS-CoV 2 infected patients [48]. However, the combination therapy of lopinavir and ritonavir was also stopped early in 13 patients (total recruitment 99 patients) due to associated gastrointestinal adverse events [48].

The severity of antiviral therapy adverse events has led researchers to explore the potential of macro-, micro- and phytonutrients that can potentially promote an immune response and suppress viral induced effects. Vitamins are known to modulate the host immune functions by providing anti-oxidants and anti-inflammatory activity [49, 50]. Therefore, we selected vitamins, ascorbic acid (vitamin C), cholecalciferol (vitamin D) and alpha-tocopherol (vitamin E) to investigate their potential interactions with the enzyme SARS-CoV-2 3CL^pro^. Our docking results interestingly, showed that vitamin D has the lowest binding energy and K*i* (− 7.75 kcal/mol and 2.08 µM respectively) as compared to vitamin C and vitamin E. Amino acid residues Thr24, Thr26, His41 and Cys145 of SARS-CoV-2 3CL^pro^ showed hydrogen bond formation with vitamin D (Fig. 7d). Amino acid Thr is extensively involved in intracellular signalling changes through phosphorylation changes, and here we observed that cholecalciferol formed a strong hydrogen bond with Thr residues and could potentially block the phosphorylation of Thr residue in SARS-CoV-2 3CL^pro^ enzyme. There is evidence that serious SARS-CoV-2 infected cases have reported severe vitamin D deficiency and thus therapeutic concentrations of this molecule could potentially be used clinically in SARS-CoV-2 cases [51, 52].

## Discussions

The novel coronavirus termed “nCovid-19” is now known as the third large-scale epidemic coronavirus introduced into the human population in the twenty-first century. At the time of writing, more than 3.67 million confirmed cases globally, with nearly 250,000 deaths had been reported by WHO. Clinically, nCovid-19 is similar to SARS regarding its presentation, however the sheer capacity and speed of which nCovid-19 has spread to global pandemic levels have left researchers asking what makes this outbreak so similar in presentation, yet so different in its virulence to previous coronaviruses. Genome sequence analysis has looked to investigate similarities in the phylogeny of SARS-CoV-2, which like SARS and MERS, have now placed it in the betacoronavirus genus [53]. The known severe and often fatal pathogenicity of betacoronaviruses has been highlighted in these previous epidemics and has reported higher transmission and pathogenicity than the milder and lesser known a-CoVs, which are often compared to the common cold [54]. Our study further compares the similarities between SARS-CoV and SARS-CoV-2 using Clustal Omega alignment to show that of 918 SARS nucleotides, there was a similarity of approximately 95%. Furthermore, we report high amino acid sequence identity in both SARS-CoV and SARS-CoV-2 main protease 3CL^pro^, which regulates coronavirus replication complexes [55]. Such highly conserved regions in both catalytic sites and the substrate binding regions of the enzymes has also been validated previously in studies by Huang et al. and Muramatsu et al. [44, 45]. While this region provides an attractive target for anti-viral drug design, it also can begin to elucidate on viral origins and uncover its ease in transmission.

Based on more recent virus genome sequencing results and evolutionary analysis, the origins and transmission of nCovid-19 have uncovered bats as the natural host of the virus origins [42]. As such, studies earlier this year queried the unknown intermediate host between bats and humans, and recent studies have pointed this to pangolins [41, 42]. To determine the extent of the evolutionary relationship between bat-CoV, Pangolin-CoV and SARS-CoV-2, we corroborate that based on the nucleotide sequences of the whole-genome sequence, bat-SARS-CoV and SARS-CoV-2 are grouped together and share > 96% similarity, with Pangolin-SARS-CoV as the closest evolutionary ancestor [41, 42]. Furthermore, we report that in isolates of human Wuhan SARS-CoV-2 there is an 85.98% similarity in identity to Pangolin-SARS-CoV, which suggests that Pangolin may be associated with the evolution of subsequent outbreaks of COVID-19.

Regarding nCovid-19 and its similarity in transmission to SARS-CoV, recent studies have also demonstrated that transmission occurs via the receptor angiotensin-converting enzyme 2 (ACE2) [42]. This may indicate why SARS-CoV-2 has often led to severe and in many cases fatal respiratory tract infections, like its two SAR-CoV predecessors. Since the SARS-CoV epidemic of 2002 was also known to use the ACE2 receptor to infect humans [56]. Bronchoalveolar lavage fluid taken from nCovid-19 patients have shown that ACE2 is widely distributed in the lower respiratory tracts of humans [42]. Furthermore, the virion S-glycoproteins expressed on the surface of coronaviruses adhere to ACE2 receptors on human cells [57]. This location provides a target for uncovering the mechanistic insights into the severity of the disease and how this region has assisted in the zoonosis of SARS-CoV-2 specifically. Additionally, mutations in the genomic structure of SARS-CoV-2 also might elucidate on the aggressiveness and pathogenicity of the viruses, which may in turn help to explain why some strains are evolutionarily much more virulent and contagious. Angeletti et al. have described mutations in the endosome-associated-protein-like domain of the nsp2 and nsp3 proteins, the former possibly accounting for the high virulence and contagion, while the latter suggesting a mechanism that differentiates nCovid-19 from SARS-CoV [58]. Our studies build on this knowledge and assist to begin to identify the sub-clinical causes for the virulence and unique pandemic pattern of this outbreak by identifying the evolving mutations from region to region. Additionally, previous studies by Pachetti et al. have reported novel recurrent mutations of the SARS-Cov-2, and our study corroborates these mutations in South America and Africa regions [43].

Drug discovery and vaccine development against SARS-CoV-2 infection require time and lengthy processes, however drug repurposing represents an alternative strategy in the current scenario. Some of these antivirals are currently being used clinically in SARS-CoV-2 treatment, including lopinavir [59], ritonavir [60], remdesivir [61], and oseltamivir [62]. However, in the clinical setting, lopinavir/ritonavir, a 3CL^pro^ and RdRp inhibitors, showed no benefit in Covid-19 adult patients [48]. The double point mutation in RdRp gene identified in our study can potentially lead to a drug-resistance event. Moreover, other classes of drugs, such as chloroquine and hydroxychloroquine have shown antiviral properties by blocking viral entry into cells by inhibiting glycosylation of host receptors [63]. We observed no differences in the SARS-CoV-2 main proteinase, 3CL^pro^ genome sequences, but important differences in SARS-CoV-2 3CL^pro^ with SARs-CoV protein, underlining the extreme need for identification of inhibitors to target the viral life cycle. It is not known whether these mutations induce any alterations in the gene transcription or localisation of affected proteins which can be investigated in near future using biochemical and immunological approaches [64, 65].

## Conclusions

Various theories have been proposed regarding the origin of highly virulent SARS-CoV-2 particle. Our analysis shows that Bat-SARS-CoV shares > 90% similarity with the SARS-CoV-2, however it is possible that the bat coronavirus infected another “intermediate host”, such as Pangolin, which subsequently transmitted the virus to humans. Pangolin isolates do share sequence identity with SARS-CoV-2 genomes and could be an intermediate host. We identified novel mutation hotspot regions from South American and African isolates of SARS-CoV-2 genome sequences. Interestingly, double point mutations in RdRp at position 14,805 and 14,808 and triple point mutations in nucleocapsid protein at position 28,881, 28,882 and 28,883 were identified in both South American and African genomic sequences, suggesting the vulnerability of these genetic loci to undergo change. In addition, a novel mutation pattern specifically oriented towards nucleocapsid phosphoprotein in both South American and African sequences was noted while novel ORF3a and RdRp specific variants were observed particularly from African genomic sequences. The potential effects of double and triple point mutations on translated proteins and the virulence of SARS-CoV-2 requires further investigations. SARS-CoV-2 main proteinase, 3CL^pro^ genome was observed to be conserved across all collected genomic sequences. Despite significant similarities in the SARS-CoV 3CL^pro^ structure with SARS-CoV protein, SARS-CoV-2 3CL^pro^ revealed certain key differences, which highlight the extreme need for identification of novel mechanism-based drugs to target the virus processing. Repurposed drugs including natural flavonoids and bioflavonoids, antimalarial, antiviral and vitamins-based compounds have previously been shown to be beneficial in several viral infections and outbreaks. The novel data generated from this study enhances our knowledge of the fine molecular differences that differentiate SARS-CoV-2 virus SARS-CoV. It also highlights the emerging variations in the viral genome across different populations as the virus evolves to local genetic and environmental factors. These findings will likely play a key role in the development of mechanism-based and targeted therapeutic strategies to treat SARS-CoV-2 infection and reduce its virulence.

## Supplementary information

**Additional file 1: Table S1.** Acknowledgement table containing information about authors, originating, and submitting laboratories of the sequences deposited to GISAID database.

**Additional file 2: Table S2.** SARS-CoV and SARS-CoV-2 sequence alignment of 3CLPro shares around 95% similarity.

## Data Availability

GISAID database (https://www.gisaid.org/), SARS-CoV-2 isolate Wuhan-Hu-1, complete genome (https://www.ncbi.nlm.nih.gov/nuccore/1798174254)

## Abbreviations

SARS-CoV-2: Severe acute respiratory syndrome coronavirus (nCovid-19)
3 CLPro: 3-Chymotrypsin-like cysteine protease
WHO: World Health Organisation
nCoV: Novel coronavirus
ORF: Open reading frame
PDB: Protein Data Bank
GISAID: The Global Initiative on Sharing Avian Influenza Data
MERS: Middle East respiratory syndrome
RBD: Receptor binding domain
RdRP: RNA-dependent RNA polymerase
nt: Nucleotide
MSA: Multiple sequence alignment
iTOL: Interactive tree of life
AM1: Austin model-1
RMS: Root mean square
MOPAC: Molecular orbital package
DS: Discovery studio
nsp: Non-structural protein
