# Supplementary material for: Evolving geographic diversity in SARS-CoV2 and in silico analysis of replicating enzyme 3CLpro targeting repurposed drug candidates
Source: J Transl Med. 2020 Jul 9;18:278. doi: 10.1186/s12967-020-02448-z (PMC7344048; doi:10.1186/s12967-020-02448-z)
Supplement: Supplementary file 2 — Additional file 2: Table S2. SARS-CoV and SARS-CoV-2 sequence alignment of 3CLPro shares around 95% similarity. [file 12967_2020_2448_MOESM2_ESM.docx]

**Additional file 2: Table S2** SARS-CoV and SARS-CoV-2 sequence alignment of 3CLPro shares around 95% similarity

NC_045512 agtggttttagaaaaatggcattcccatctggtaaagttgagggttgtatggtacaagta 60

NC_004718 agtggttttaggaaaatggcattcccgtcaggcaaagttgaagggtgcatggtacaagta 60

*********** ************** ** ** ******** ** ** ************

NC_045512 acttgtggtacaactacacttaacggtctttggcttgatgacgtagtttactgtccaaga 120

NC_004718 acctgtggaactacaactcttaatggattgtggttggatgacacagtatactgtccaaga 120

** ***** ** ** ** ***** ** * *** * ****** *** ************

NC_045512 catgtgatctgcacctctgaagacatgcttaaccctaattatgaagatttactcattcgt 180

NC_004718 catgtcatttgcacagcagaagacatgcttaatcctaactatgaagatctgctcattcgc 180

***** ** ***** * ************** ***** ********* * ********

NC_045512 aagtctaatcataatttcttggtacaggctggtaatgttcaactcagggttattggacat 240

NC_004718 aaatccaaccatagctttcttgttcaggctggcaatgttcaacttcgtgttattggccat 240

** ** ** **** ** * ** ******** *********** * ******** ***

NC_045512 tctatgcaaaattgtgtacttaagcttaaggttgatacagccaatcctaagacacctaag 300

NC_004718 tctatgcaaaattgtctgcttaggcttaaagttgatacttctaaccctaagacacccaag 300

*************** * **** ****** ******** * ** *********** ***

NC_045512 tataagtttgttcgcattcaaccaggacagactttttcagtgttagcttgttacaatggt 360

NC_004718 tataaatttgtccgtatccaacctggtcaaacattttcagttctagcatgctacaatggt 360

***** ***** ** ** ***** ** ** ** ******** **** ** *********

NC_045512 tcaccatctggtgtttaccaatgtgctatgaggcccaatttcactattaagggttcattc 420

NC_004718 tcaccatctggtgtttatcagtgtgccatgagacctaatcataccattaaaggttctttc 420

***************** ** ***** ***** ** *** ** ***** ***** ***

NC_045512 cttaatggttcatgtggtagtgttggttttaacatagattatgactgtgtctctttttgt 480

NC_004718 cttaatggatcatgtggtagtgttggttttaacattgattatgattgcgtgtctttctgc 480

******** ************************** ******** ** ** ***** **

NC_045512 tacatgcaccatatggaattaccaactggagttcatgctggcacagacttagaaggtaac 540

NC_004718 tatatgcatcatatggagcttccaacaggagtacacgctggtactgacttagaaggtaaa 540

** ***** ******** * ***** ***** ** ***** ** **************

NC_045512 ttttatggaccttttgttgacaggcaaacagcacaagcagctggtacggacacaactatt 600

NC_004718 ttctatggtccatttgttgacagacaaactgcacaggctgcaggtacagacacaaccata 600

** ***** ** *********** ***** ***** ** ** ***** ******** **

NC_045512 acagttaatgttttagcttggttgtacgctgctgttataaatggagacaggtggtttctc 660

NC_004718 acattaaatgttttggcatggctgtatgctgctgttatcaatggtgataggtggtttctt 660

*** * ******** ** *** **** *********** ***** ** ***********

NC_045512 aatcgatttaccacaactcttaatgactttaaccttgtggctatgaagtacaattatgaa 720

NC_004718 aatagattcaccactactttgaatgactttaaccttgtggcaatgaagtacaactatgaa 720

*** **** ***** *** * ******************** *********** ******

NC_045512 cctctaacacaagaccatgttgacatactaggacctctttctgctcaaactggaattgcc 780

NC_004718 cctttgacacaagatcatgttgacatattgggacctctttctgctcaaacaggaattgcc 780

*** * ******** ************ * ******************** *********

NC_045512 gttttagatatgtgtgcttcattaaaagaattactgcaaaatggtatgaatggacgtacc 840

NC_004718 gtcttagatatgtgtgctgctttgaaagagctgctgcagaatggtatgaatggtcgtact 840

** *************** * ** ***** * ***** ************** *****

NC_045512 atattgggtagtgctttattagaagatgaatttacaccttttgatgttgttagacaatgc 900

NC_004718 atccttggtagcactattttagaagatgagtttacaccatttgatgttgttagacaatgc 900

** * ***** ** * *********** ******** *********************

NC_045512 tcaggtgttactttccaa 918

NC_004718 tctggtgttaccttccaa 918

** ******** ******
